# Neglected Pediatric Osteochondral Fracture Dislocation of the Patella

**DOI:** 10.1155/2019/2904782

**Published:** 2019-10-21

**Authors:** Ta-Li Hsu, Shang-Ming Lin, Chih-Hung Chang, Tsung-Yu Lan

**Affiliations:** ^1^Department of Orthopedic Surgery, Far Eastern Memorial Hospital, New Taipei City, Taiwan; ^2^Department of Materials and Textiles, Oriental Institute of Technology, New Taipei City, Taiwan; ^3^Yuan Ze University Graduate School of Biotechnology and Bioengineering, Taiwan

## Abstract

Pediatric osteochondral fracture dislocation of the patella is sometimes difficult to diagnose on the basis of physical examination or plain film radiography. Magnetic resonance imaging plays an important role in its early diagnosis, and early treatment can prevent damage to the articular cartilage as well as decrease the dislocation rate. Currently, many treatment choices have been reported with good results, but there is no consensus on which treatment option may lead to the best outcome. Herein, we describe the case of a 14-year-old girl with neglected osteochondral fracture dislocation of the patella. The outcome was optimal on the basis of a 2-year postoperative follow-up; thus, we believe that fixation with headless screws is a simple and effective method if the fracture fragment is large enough.

## 1. Introduction

Osteochondral fractures of the femur or patella associated with patellar dislocation are more frequently noted in the pediatric population than in adults and may be accompanied by a spectrum of soft tissue injuries [1, 2]. Because of neglect in the initial phase [2], repositioning of these fractures in skeletally immature patients is important to improve healing capacity [2, 3] and to prevent secondary osteoarthritis with pain and functional limitation. Many treatment options have been developed recently without consensus on which treatment option may lead to the best outcome [3]. Herein, we describe a case of neglected osteochondral fracture dislocation of the patella that was successfully treated with open reduction and internal fixation on the basis of clinical and radiographic results.

## 2. Case Presentation

A 14-year-old girl without any previous clinical history presented with a painful, swollen right knee joint after playing volleyball. She was first treated conservatively for knee sprain at another institute, but the pain persisted. She was brought to our clinic for a second opinion 2 weeks after the injury.

**Figure 1:**
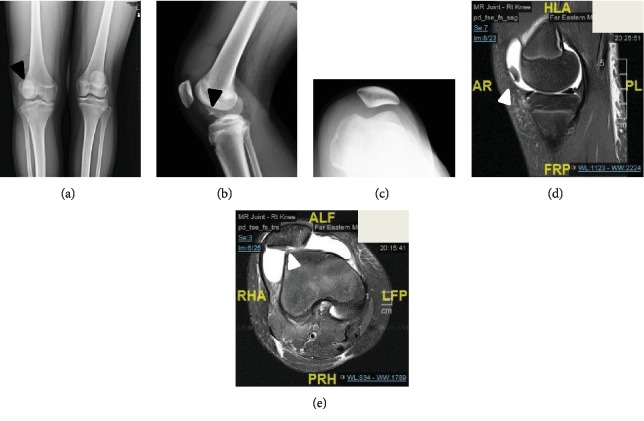
Preoperative radiographs and magnetic resonance images. (a–c) Preoperative standard radiographs of the patella. (d, e) Preoperative magnetic resonance images. The arrowheads indicate the neglected osteochondral fracture dislocation of the patella.

The physical examination of the right knee showed loss of full extension, swelling during the range of motion, and tenderness. Initial radiographs showed lateral subluxation of the right patella with one fragment in the knee joint (Figures 1(a)–1(c)). Magnetic resonance imaging (MRI) demonstrated an osteochondral defect over the central aspect of the patella with a large loose body (Figures 1(d) and 1(e)). Surgery was suggested.

**Figure 2:**
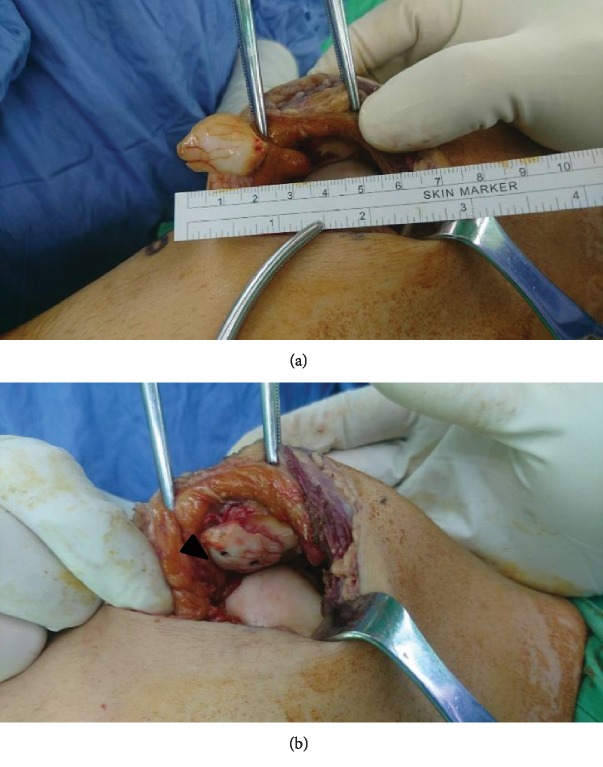
(a) Intraoperative view of osteochondral fracture dislocation of the patella. (b) The arrowhead indicates the fractured fragment. The fragment is perfectly reduced and fixed by two cannulated screws. The screw head is totally embedded.

On the third posttrauma week, open reduction and internal fixation were performed. Under general anesthesia with the patient in the supine position, we performed a medial parapatellar arthrotomy to approach the fractured patella. The patella was flipped laterally, and the articular surface of the patella was exposed. Debridement and irrigation of the fracture site with normal saline were conducted. Finally, reduction was performed, and the fracture was fixed with two headless screws (Acutrak®, Acumed, Beaverton, Oregon) (Figure 2(a)). The medial patellofemoral ligament was also repaired. Intraoperative findings revealed the osteochondral fragment (30 × 25 × 10 mm^3^) from the central aspect of the patella (Figure 2(b)), an intra-articular loose body, hemarthrosis, and rupture of the medial patellofemoral ligament, which caused lateral subluxation of the patella. The calculated surgical time from incision to wound closure was 1 hour.

Postoperatively, partial weight bearing was immediately allowed and a dynamic functional knee brace was applied. The knee was fixed in full extension for 2 weeks, gradual knee flexion was allowed, and physical therapy was prescribed.

**Figure 3:**
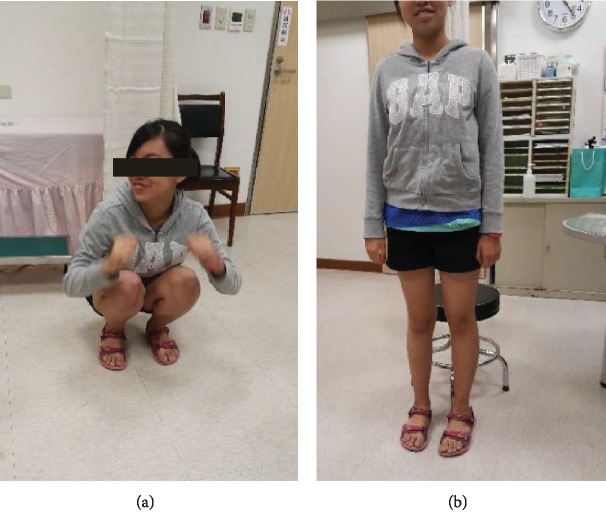
Postoperative status at 3 months. (a, b) The patient can do squats and sports activities as well as she could before the injury.

**Figure 4:**
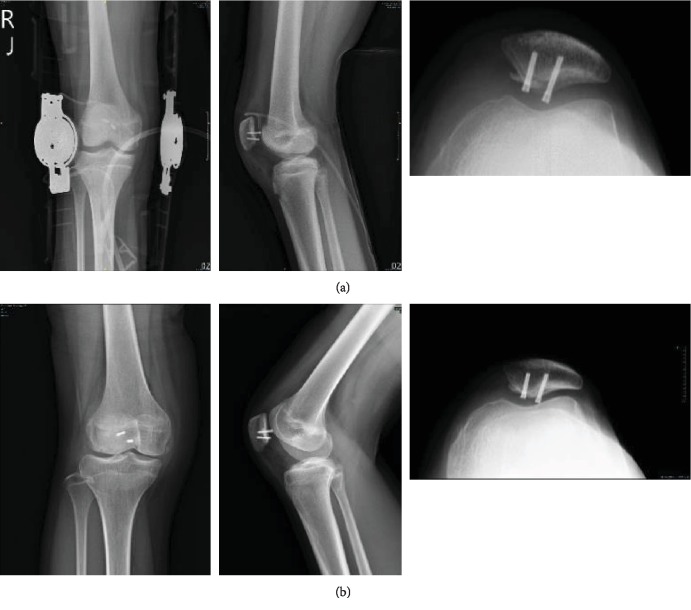
(a) Postoperative standard radiographs at the 1-week follow-up. Note that the dislocation and articular surface are corrected. (b) Postoperative standard radiograph showing a healed fracture site and the implant in situ at 9 months.

After 2 months postoperatively, the patient showed good patella gliding without pain. She could do squats and sports activities as well as she could before the injury (Figures 3(a) and 3(b)). Follow-up radiographs also demonstrated healing of the osteochondral fracture (Figures 4(a) and 4(b)). After 1 year, implant irritation was noted, and implant removal was suggested.

**Figure 5:**
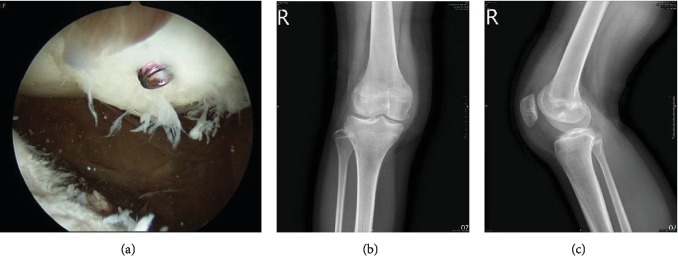
(a) Postoperative second-look arthroscopy at 1-year follow-up showing blurring of the cartilage and metallosis but no obvious degenerative changes in the cartilage. (b, c) Postoperative radiograph shows the healed fracture.

Second-look arthroscopy was performed and showed resorption of the cartilage over the screw head. The screw head and some metallosis were found. There were no significant degenerative changes in the cartilage (Figures 5(a)–5(c)). After removal of the implant, her discomfort disappeared.

## 3. Discussion

Although the exact prevalence of osteochondral fractures is still unknown [1, 2], they still account for 30 to 50% of the acute patellar dislocation rate [2]. The frequent delay in diagnosis naturally affects the healing capacity after repositioning these lesions. The most affected area is located around the medial patellar facet or at the lateral side of the distal femur [4, 5]. To date, the diagnosis of osteochondral fracture dislocation of the patella is difficult to establish on the basis of a physical examination or plain film radiography. Negative radiography results have been reported in about 36% of children with osteochondral fracture [6]. If the patient has any complaint of acute knee pain with or without a history of trauma that is accompanied by knee effusion and major loss of mobility, patellar dislocation associated with osteochondral defect should be highly suspected. Besides, intra-articular injury is highly suspected when hemarthrosis is observed following knee trauma [4, 7, 8]. The correct diagnosis may be very difficult to make without MRI or arthroscopic examination [2–4, 8].

Timely diagnosis is key to preventing damage to the articular cartilage because the development of fibrous tissue at the fracture side will affect proper reduction [7]. The redislocation rate will also increase if it is left untreated, as observed by Hawkins et al. [5, 9]. If the osteochondral fragments are too small to be fixed to the fracture site, removal of the fragment, drilling, microfracture, and chondrocyte implantation should be considered. To avoid possible damage to the cartilage and achieve rigid fixation, fragments smaller than 2 cm^2^ should be excised or managed nonoperatively for equivalent or better outcomes if the osteochondral fragment does not involve a weight-bearing surface [4, 10]. However, if the fragment is big enough, many other treatment choices are available to fix osteochondral fractures of the patella, including replacement of the native fragment with internal fixation, resorbable implant fixation, crossing suture technique, allograft replacement, autograft replacement, and autologous chondrocyte transplantation [2, 11–21]. If the osteochondral lesion is <1.5 cm^2^, osteochondral cylinder transplantation is recommended, and if the lesion is >1.5 cm^2^, autologous chondrocyte implantation plus subchondral bone grafting should be considered, according to Salzmann et al. [10]. The goal is to reproduce a smooth gliding articular surface of the hyaline cartilage, which may prevent secondary osteoarthritis with pain and functional limitation [3]. To date, there is still no consensus on which treatment option may lead to the best outcomes. For smaller osteochondral fragments, the crossing suture technique with ultrabraid (Smith & Nephew, Memphis, TN) is a treatment choice [6]. Although good long-term functional results have been mentioned with the use of bioabsorbable ultra-high strength poly(L-lactide) pins or absorbable sutures (Vicryl 2, Ethicon, Somerville, NJ) [3, 13], implant breakage, foreign body reaction, and aseptic synovitis have been reported [6, 22]. A two-component fibrin sealant (adhesive system of fibrin) and butyl-2-cyanoacrylate tissue glue (first synthesized in 1949) were both reported to yield good results if the osteochondral fragments were large enough or too thin to be secured with anchors or Herbert screws [19, 20]. Headless cannulated compression screws were also successfully used by Rae and Khasawneh in 1988 to fix fragments that were large enough [16, 17]. Local friction and irritation are thought to be prevented when the screws are completely embedded in the cartilage and bone. Embedding the screw at a depth of 3 mm has been recommended in order to achieve peak compression strength [17]. After a recent review of the literature, we found that no study has recommended which kind of fragment size is better for headless cannulated compression screw fixation. On the basis of our experience, we recommend the use of a fragment size larger than 3 cm^2^ for headless cannulated compression screw fixation, as this results in no degenerative changes in the cartilage and a good clinical outcome.

The main limitation of the present study is the small number of patients. Further studies of this technique with more patients are needed to clarify the outcome of this surgical technique.

Early MRI plays an important role in making the correct diagnosis of neglected osteochondral fracture dislocation of the patella. Our case was initially diagnosed as an anterior cruciate ligament avulsion fracture and then diagnosed as neglected osteochondral fracture dislocation of the patella on the basis of the MRI findings. Treatment of osteochondral fracture dislocation of the patella is sometimes challenging. However, internal fixation with headless screws is an effective method for a large osteochondral fragment. The follow-up result was clinically good in our case, but second surgery to remove the implant is sometimes necessary.
